# JA-Induced Endocytosis of AtRGS1 Is Involved in G-Protein Mediated JA Responses

**DOI:** 10.3390/ijms20153779

**Published:** 2019-08-02

**Authors:** Li Li, Bodan Su, Xueying Qi, Xi Zhang, Susheng Song, Xiaoyi Shan

**Affiliations:** 1 College of Biological Sciences and Biotechnology, Beijing Forestry University, Beijing 10083, China; 2 Beijing Key Laboratory of Plant Gene Resources and Biotechnology for Carbon Reduction and Environmental Improvement, College of Life Sciences, Capital Normal University, Beijing 100048, China

**Keywords:** heterotrimeric G proteins, AtRGS1, jasmonates, endocytosis, diffusion dynamics

## Abstract

*Arabidopsis* heterotrimeric G proteins regulate diverse plant growth and defense processes by coupling to 7TM AtRGS1 proteins. Although G protein mutants display alterations in response to multiple plant hormones, the underlying mechanism by which G proteins participate in the regulation of hormone responses remains elusive. Here, we show that genetic disruption of Gα and Gβ subunits results in reduced sensitivity to JA treatment. Furthermore, using confocal microscopy, VA-TIRFM, and FRET-FLIM, we provide evidence that stimulation by JA induces phosphorylation- and C-terminus-dependent endocytosis of AtRGS1, which then promotes dissociation of AtRGS1 from AtGPA1. In addition, SPT analysis reveals that JA treatment affects the diffusion dynamics of AtRGS1 and AtRGS1-ΔCt. Taken together, these findings suggest that the JA signal activates heterotrimeric G proteins through the endocytosis of AtRGS1 and dissociation of AtRGS1 from AtGPA1, thus providing valuable insight into the mechanisms of how the G protein system perceives and transduces phytohormone signals.

## 1. Introduction

Jasmonates (JAs), which include jasmonic acid and its oxylipin derivatives, are synthesized from the octadecanoid/hexadecanoid pathways and are widely distributed throughout the plant kingdom [1]. As one of the important plant hormones, JAs function as growth regulators and defense signals that control various plant developmental processes, such as root growth, anthocyanin accumulation, male fertility, and leaf senescence, as well as mediate plant responses to abiotic and biotic stresses, including insect attack, UV damage, pathogen infections, and wounding [2]. The jasmonate ZIM-domain proteins (JAZs) that consist of 12 members act as repressors to negatively regulate diverse JA responses by directly binding to downstream transcriptional factors (TFs) or interactions with the co-repressor TOPLESS (TPL) via the novel interactor of JAZ (NINJA) [3]. Upon perception of a JA signal, the F-box protein coronatine insensitive 1 (COI1) forms an SCF^COI1^ complex with *Arabidopsis* SKP1 homologue 1 (ASK1)/ASK2, AtCullin1, and *Arabidopsis* Ring-box 1 (AtRbx1) to recruit JAZs for ubiquitination and degradation through the 26S proteasome, which subsequently activates downstream TFs [3].

Signal transduction through a heterotrimeric G protein complex, classically consisting of Gα, Gβ, and Gγ subunits, is an essential plasma membrane (PM) signaling pathway in most eukaryotes [4,5]. In animal cells, the heterotrimeric G proteins are directly regulated by seven transmembrane (7TM) G protein-coupled receptors (GPCRs) that could catalyze the nucleotide exchange from guanosine triphosphate (GTP) to guanosine diphosphate (GDP) on the Gα subunit upon stimulation [6]. Thus, GTP-bound Gα and Gβγ dimers are released to activate downstream effectors, thereby relaying diverse intracellular signals. In *Arabidopsis*, this complex comprises one canonical Gα subunit (AtGPA1), one Gβ subunit (AGB1), and one of three Gβ subunits (AGG1, 2, and 3) [7,8,9,10]. In contrast to animal alpha subunits, AtGPA1 spontaneously undergoes the GDP- and GTP-exchange cycle. AtRGS1, a GPCR-like N-terminal 7TM fused to a regulator of G-protein signaling (RGS) protein, keeps the complex in the inactive state by promoting GTP hydrolysis of AtGPA1 [11,12]. d-glucose or other stimuli can induce the dissociation of AtGPA1 from AtRGS1 to maintain its self-active state and trigger effector activities [13]. Genetic analysis of loss-of-function G protein mutants in *Arabidopsis* and rice support the fundamental roles of heterotrimeric G proteins in various biological processes, including morphological development from seed germination to silique development, response to glucose, light stimuli and hormones, stomatal movements, and ion channel regulation, as well as innate immunity [13].

Endocytosis of the 7TM receptors in mammals and plants is considered to be a vital step in the regulation of G protein signaling. In animals, GPCRs are phosphorylated and then endocytosed through a clathrin-dependent pathway to desensitize ligand stimulation [6]. However, phosphorylated AtRGS1 protein is internalized to release its inhibition upon AtGPA1 self-activation, permitting sustained activation of G protein signaling. In the presence of glucose, WITH NO LYSINE kinases (WNKs) can phosphorylate AtRGS1 at the C-terminal domain for endocytosis, which is critical to the activation of G protein-mediated sugar signaling and cell proliferation [14,15]. After induction of a conserved 22-amino acid domain in the N-terminus of flagellin (flg22), AtRGS1 is phosphorylated by BRI1-associated kinase 1 (BAK1) at its C-terminal tail and then internalized [16,17]. Thus, the G protein complex is physically uncoupled from its repressor, and then interacts with its effectors to regulate reactive oxygen species (ROS) production and calcium release [17,18,19]. In addition, sodium-induced activation of G signaling via AtRGS1 endocytosis plays a key role in plant responses to salt stress [20].

In the present study, we found that the loss of Gα and Gβ subunits conferred hyposensitivity to JA signaling. Furthermore, we demonstrated that JA induced the endocytosis of AtRGS1 in a phosphorylation- and C-terminus-dependent manner, which in turn led to the dissociation of AtRGS1 from AtGPA1 for activation of downstream signaling. In addition, the diffusion dynamics of AtRGS1 and C-terminus-truncated AtRGS1 proteins changed upon JA treatment. Our analyses propose a possible role for JA-induced endocytosis of AtRGS1 in G-protein-mediated JA responses and support the hypothesisthat plant hormone signaling might be relayed, in part, by the AtRGS1-G protein pathway.

## 2. Results and Discussion

### 2.1. The Heterotrimeric G Protein Complex Is Involved in JA Signaling

Considering that G protein mutants exhibit various phenotypes during growth and in defense responses, it is possible that the heterotrimeric G protein complex might be involved with JA signaling, which regulates plant development and immunity. To gain insights into the relationship between G proteins and JA signaling, we subjected wild-type (WT, Col-0), Gα-deficient mutant (*gpa1-4*), Gβ-deficient mutant (*agb1-2*), and double mutant *gpa1-4/agb1-2* to a series of JA response assays.

**Figure 1:**
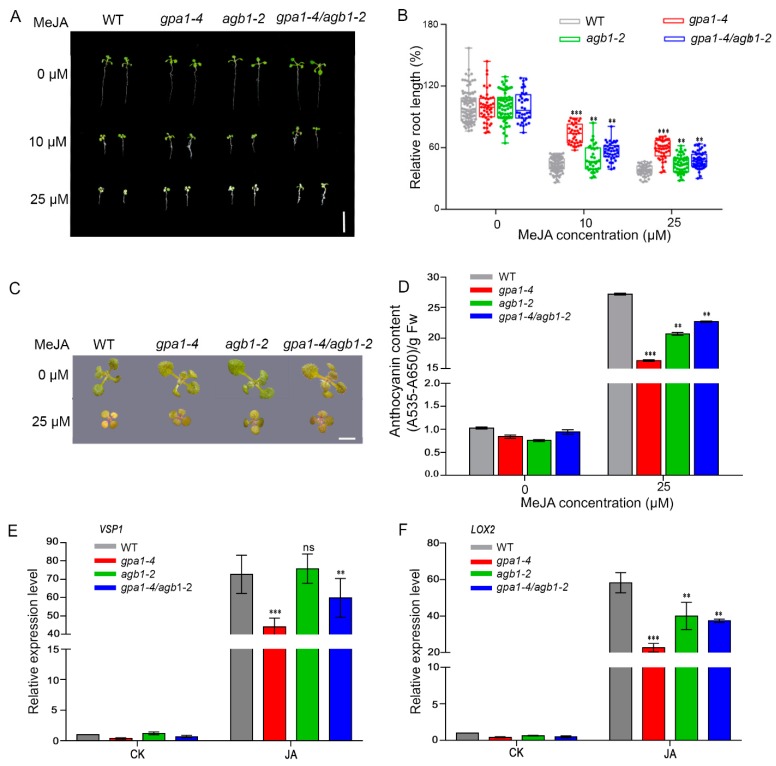
The heterotrimeric G protein complex is involved in JA signaling. (**A**) Root phenotypes of 9-day-old seedlings of WT, *gpa1-4*, *agb1-2*, and *gpa1-4/agb1-2* grown on MS medium containing indicated concentrations of MeJA. Bar = 1 cm. (**B**) Relative root length of 9-day-old seedlings of WT, *gpa1-4*, *agb1-2*, and *gpa1-4/agb1-2* grown on MS medium containing indicated concentrations of MeJA. Data shown are from 34 to 104 plants. Significant differences are denoted by asterisks (** *p* < 0.01, *** *p* < 0.001, Student’s *t* test). (**C**) Phenotype of 11-day-old seedlings of WT, *gpa1-4*, *agb1-2*, and *gpa1-4/agb1-2* grown on MS medium containing indicated concentrations of MeJA. Bar = 1 cm. (**D**) Anthocyanin contents of 11-day-old seedlings of WT, *gpa1-4*, *agb1-2*, and *gpa1-4/agb1-2* grown on MS medium containing indicated concentrations of MeJA. Error bars represent SD (*n* = 3). Significant differences are denoted by asterisks (** *p* < 0.01, *** *p* < 0.001, Student’s *t* test). (**E**,**F**), Relative expression level of *VSP1* (**E**) and *LOX2* (**F**) in WT, *gpa1-4*, *agb1-2*, and *gpa1-4/agb1-2* seedlings treated without (CK) or with 100 μM MeJA for 8 h. Error bars represent SD (*n* = 3). Significant differences are denoted by asterisks (** *p* < 0.01, *** *p* < 0.001, ns, no significant difference, Student’s *t* test).

Compared to seedlings without methyl jasmonate (MeJA) treatment, each genotype exhibited JA-induced inhibition of primary root growth when grown on MS medium with different concentrations of MeJA (Figure 1A,B). However, the stunted growth of *gpa1-4*, *agb1-2*, or *gpa1-4/agb1-2* mutants was significantly less than that of WT seedlings (73%, 50%, and 56% for *gpa1-4*, *agb1-2*, and *gpa1-4/agb1-2*, respectively, 44% for WT upon 10 μM MeJA; 58%, 44%, and 47% for *gpa1-4*, *agb1-2*, and *gpa1-4/agb1-2*, respectively, 38% for WT upon 25 μM MeJA) (Figure 1A,B). Consistent with the root length phenotype, the G protein mutants were also hyposensitive to JA-induced anthocyanin accumulation. The anthocyanin content of WT plants was 1.7-, 1.3-, and 1.2-fold greater than that of *gpa1-4*, *agb1-2*, and *gpa1-4/agb1-2*, respectively, in response to JA induction (Figure 1C,D). Moreover, JA treatment could upregulate the expression of *VSP1* and *LOX2* in each genotype, whereas the expression level in *gpa1-4* and *gpa1-4/agb1-2* mutants was significantly less than the WT seedlings (Figure 1E,F). For example, *VSP1* expression was reduced by 36% and 14% in the *gpa1-4* and *gpa1-4/agb1-2* mutant in comparison to that in the WT seedling, respectively (Figure 1E). The JA-induced expression level of *LOX2* was also noticeably decreased in the *agb1-2* plants, which was about half of that in the WT seedlings (Figure 1F). However, the *VSP1* induction pattern was almost identical in WT and *agb1-2* mutants (Figure 1E).

Collectively, knockout mutants of *AtGPA1* and *AGB1* display and share reduced sensitivity to JA in root growth, anthocyanin accumulation, and *LOX2* gene expression (Figure 1). These findings suggest that both Gα and Gβ subunits likely function in some, but not all, JA responses as positive regulators.

### 2.2. JA Induces AtRGS1 Endocytosis and Dissociation from AtGPA1

**Figure 2:**
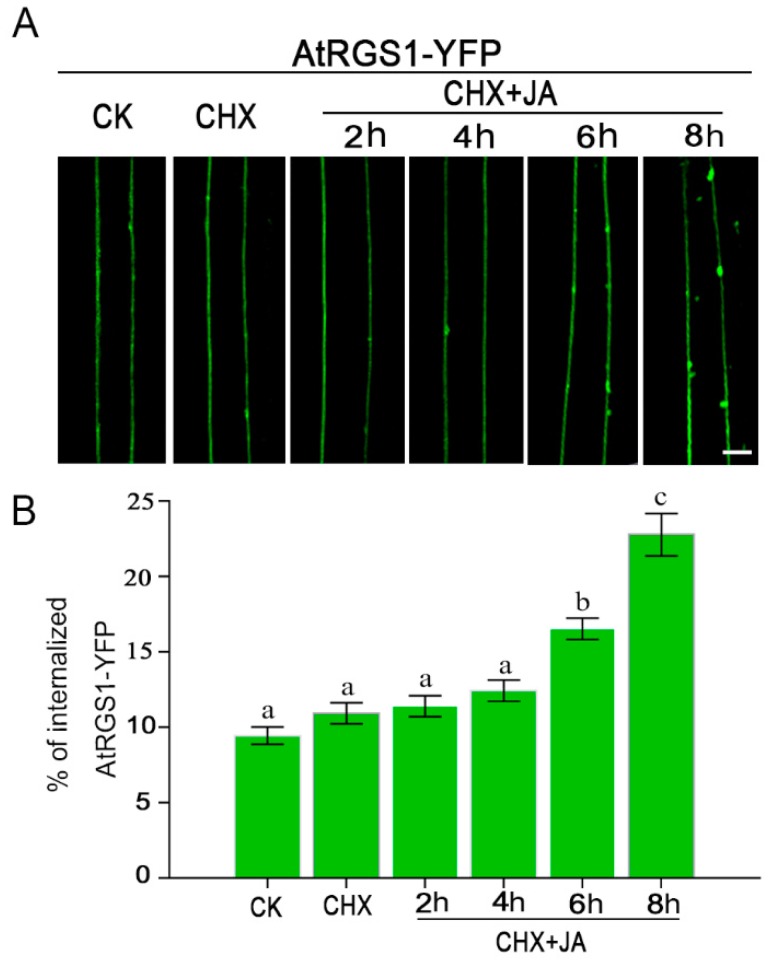
JA induces the endocytosis of AtRGS1. (**A**) Confocal images of AtRGS1-YFP localization without treatment (CK), with CHX treatment and with MeJA plus CHX pretreatment for indicated times in an *Arabidopsis* hypocotyl epidermal cell. Bar = 5 μm. (**B**) Quantification of the internalized AtRGS1-YFP without treatment (CK), with CHX treatment, and with MeJA plus CHX pretreatment for indicated times. Error bars represent SD (*n* = 5–8). Significant differences are denoted by letters (Duncan multiple-comparisons test; values within the column followed by the same letter show no significant difference (*p* > 0.05), whereas those with the different letter indicate significant difference (*p* < 0.05)).

One possible explanation for the involvement of the G-protein complex in JA signaling is that JA itself indirectly activates G proteins and their downstream effectors to regulate JA responses. Given that the endocytosis of AtRGS1 is a well-known reporter for G protein activation, plants expressing AtRGS1-YFP (Figure S1) were treated with 100 μM MeJA plus the protein synthesis inhibitor, cycloheximide (CHX), for different times, and AtRGS1-YFP internalization was quantitated. Under steady-state (CK) or with CHX treatment only, AtRGS1-YFP proteins were mainly localized to the PM (Figure 2A). When treated with MeJA for 2 and 4 h, no detectable change in the subcellular location of AtRGS1-YFP was observed (Figure 2A). Notably, distinct YFP-positive vesicles appeared in the cytoplasm upon MeJA treatment for 6 h or more (Figure 2A). By analyzing the percentage of AtRGS1 endocytosis, 2 and 4 h MeJA treatment (12% for both 2 and 4 h) showed no quantitative effect compared to the control conditions (10% for both CK and CHX only) (Figure 2B). A significant increase in internalization (15.8% of AtRGS1) was found at 6 h after MeJA treatment, and by 8 h, more extensive endocytosis was observed (23% of AtRGS1) (Figure 2B), suggesting a time-dependent AtRGS1 internalization upon JA treatment.

**Figure 3:**
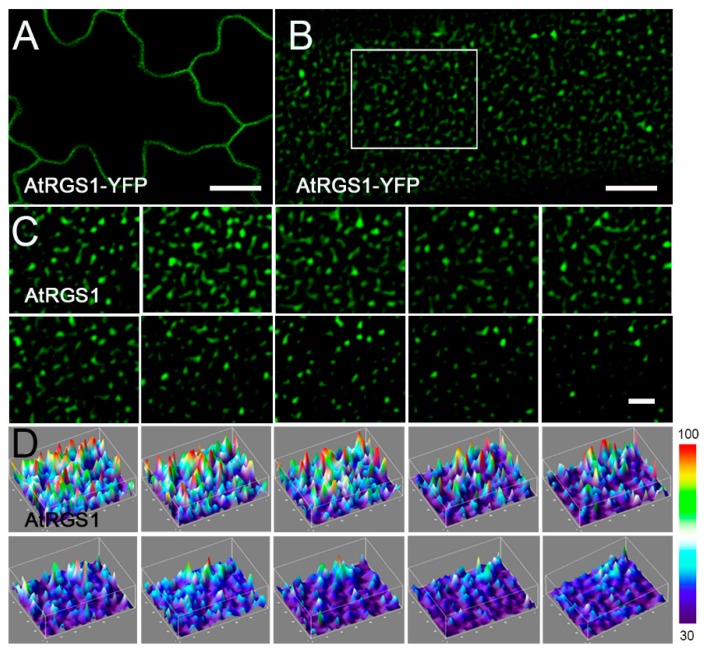
The distribution of AtRGS1 on the PM. (**A**) Confocal images of AtRGS1-YFP in *Arabidopsis* leaf epidermal cells. Bar = 5 μm. (**B**) A typical single-particle image for AtRGS1-YFP at the PM. (**C**) Sequential images of the boxed area in (**B**) with a 15 s recording in 1.5 s intervals by VA-TIRFM. Bar = 2 μm. (**D**) Three-dimensional luminance plots in (**C**) showing varied fluorescence intensity among different spots. Bar = 2 μm.

Using variable-angle total internal reflection fluorescence microscopy (VA-TIRFM), we found that AtRGS1-YFP spots localized to the PM and formed dispersed punctate structures with increased fluorescence intensities (Figure 3B). Sequential images with a 15 s recording in 0.15 s intervals showed that the individual particles remained on the PM with lateral and temporal dynamics (Figure 3C,D). To obtain more data on JA-induced endocytosis of AtRGS1, we implemented kymograph analysis to investigate the PM residence time of AtRGS1 particles in response to JA treatment. There was no significant difference between seedlings without treatment (CK, *t* = 2.06 s) and with CHX only (*t* = 2.02 s), whereas JA treatment led to significantly shorter dwell times (*t* = 1.56 s) compared to the control seedlings (CK and CHX only) (Figure 6A,B,E). These results provide evidence that JA facilitates rapid internalization of AtRGS1.

**Figure 4:**
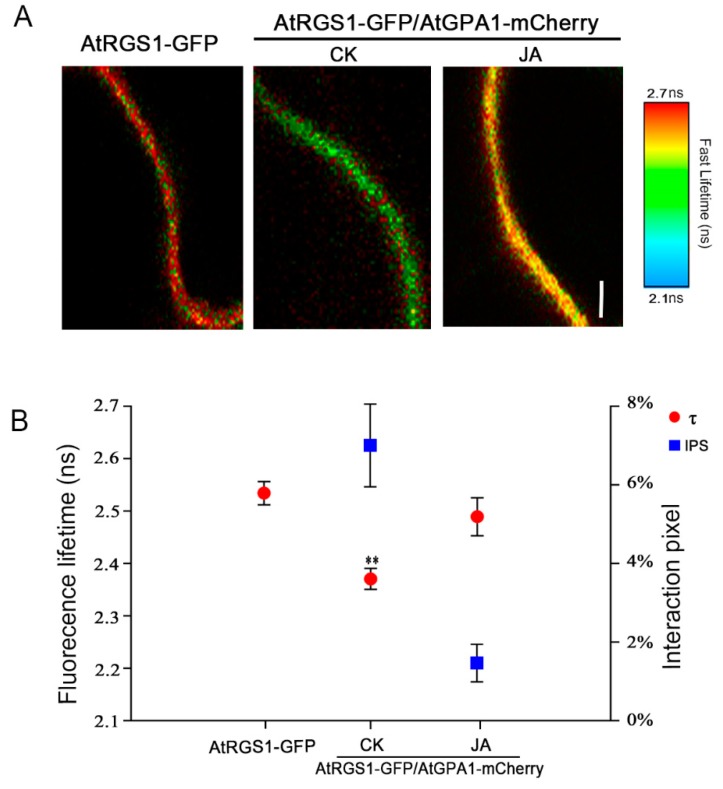
JA induces the dissociation of AtRGS1 from AtGPA1. (**A**) Fluorescence lifetime heat maps of AtRGS1-GFP alone or AtRGS1-GFP/AtGPA1-mCherry in *N. benthamiana* leaves prior and after MeJA treatment for 8 h. Bar = 10 μm. The scale varies from lowest lifetime of 2.1 s to the highest lifetime of 2.7 s. (**B**) The average fluorescence lifetime (τ) and FRET efficiency (IPS) of AtRGS1-GFP alone or AtRGS1-GFP/AtGPA1-mCherry in *N. benthamiana* leaves prior and after MeJA treatment for 8 h. Error bars represent SD (*n* = 9–12). Significant differences are denoted by letters (** *p* < 0.01, Student’s *t* test).

Endocytosis has been shown to cause physical separation of the Gα subunit from AtRGS1 for sustained G protein-dependent signaling. To test whether JA-induced endocytosis of AtRGS1 also results in its dissociation from AtGPA1, we performed forster resonance energy transfer-fluorescence lifetime imaging microscopy (FRET-FLIM) assay to analyze their interaction by determining the donor fluorescence lifetime (τ) and the FRET frequency with a higher spatial temporal accuracy in living cells. When expressed alone in *Nicotiana benthamiana* leaves, the donor AtRGS1-GFP signal exhibited a generally long fluorescence lifetime (τ = 2.53 ± 0.001 ns) as depicted by the reddish pseudo-color on the heat map, whereas a ‘blue-shift’ was observed in the AtRGS1-GFP/AtGPA1-mCherry heterologous co-expression system, indicating a strong reduction in GFP lifetime (τ = 2.36 ± 0.005 ns) (Figure 4 and Figure S2). These results confirm that AtRGS1 directly interacts with AtGPA1 under steady-state conditions. In contrast, treatment of MeJA resulted in a marked increase in GFP lifetime in the AtRGS1-GFP/AtGPA1-mCherry co-expressed leaves (τ = 2.50 ± 0.006 s) (Figure 4). Meanwhile, FRET efficiency significantly decreased from 6.8% to 1.4% (Figure 4B). These observations indicate that JA triggers the dissociation of AtRGS1 and G protein pre-formed complex.

Taken together, our findings reveal that JA can stimulate the endocytosis of AtRGS1 protein and subsequently enable AtRGS1 to move away from AtRGS1. It is reasonable to propose that other potential ligands, such as plant hormones, in addition to d-glucose and flg22, could also activate G signaling as a consequence of AtRGS1 endocytosis. Therefore, the endocytosis of AtRGS1 could be the crux of signal modulation of heterotrimeric G proteins in response to diverse stimuli.

### 2.3. The Phosphorylation and C-Terminal Domain Are Required for JA-Induced AtRGS1 Endocytosis

The C-terminal phosphorylation of AtRGS1 has been shown to play a critical role in its endocytosis. Glucose-induced endocytosis of AtRGS1 is initiated by its transphosphorylation by three WNKs at the C-terminal domain [15]. Stimulation by flg22 induces the phosphorylation of AtRGS1 by the receptor-like kinase BAK1 at the C-terminal domain, leading to its endocytosis [16,17]. Therefore, we were curious as to whether the phosphorylation and C-terminal domain of AtRGS1 are necessary for JA-induced endocytosis.

**Figure 5:**
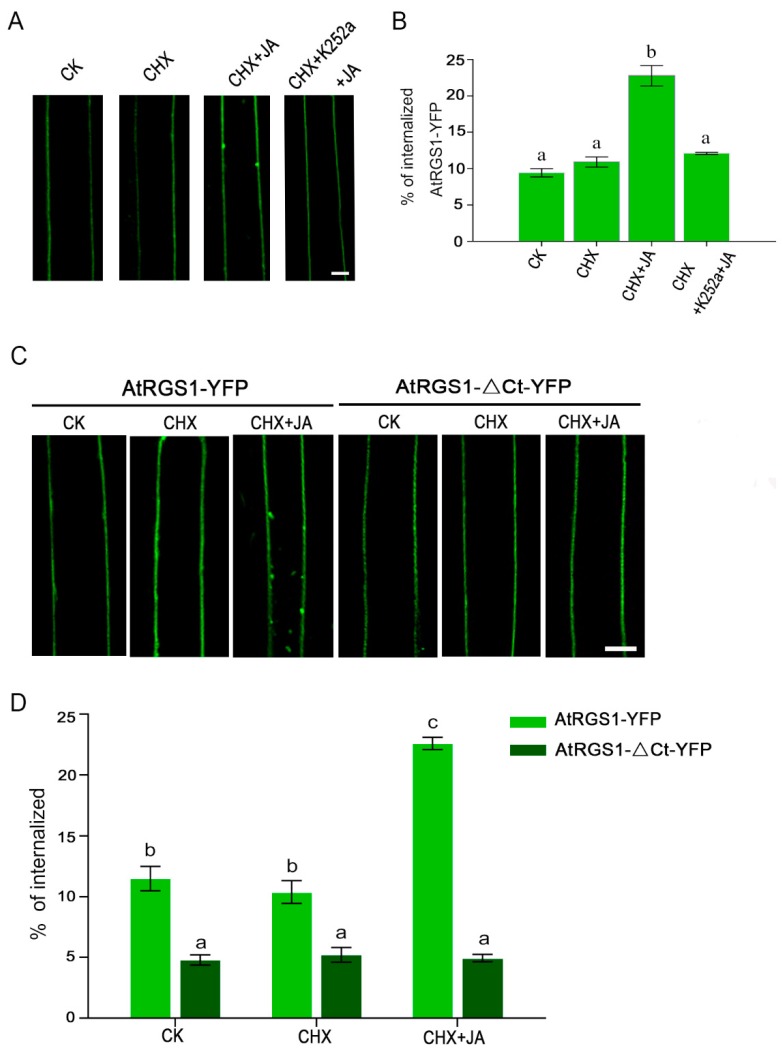
Phosphorylation and the C-terminal domain are required for AtRGS1 endocytosis. (**A**) Confocal images of AtRGS1-YFP localization without treatment (CK), with CHX treatment, with MeJA treatment for 8 h plus CHX pretreatment, and with MeJA treatment for 8 h plus CHX and K252a pretreatment in an *Arabidopsis* hypocotyl epidermal cell. Bar = 10 μm. (**B**) Quantification of the internalized AtRGS1-YFP without treatment (CK), with CHX treatment, with MeJA treatment for 8 h plus CHX pretreatment, and with MeJA treatment for 8 h plus CHX and K252a pretreatment. Error bars represent SD (*n* = 5–8). Significant differences are denoted by letters (Duncan multiple-comparisons test; values within the column followed by the same letter show no significant difference (*p* > 0.05), whereas those with different letter indicate significant difference (*p* < 0.05)). (**C**) Confocal images of AtRGS1-YFP and AtRGS1-ΔCt-YFP without treatment (CK), with CHX treatment, and with MeJA treatment for 8 h plus CHX pretreatment in an *Arabidopsis* hypocotyl epidermal cell. Bar = 10 μm. (**D**) Quantification of the internalized AtRGS1-YFP or AtRGS1-ΔCt-YFP without treatment (CK), with CHX treatment, and with MeJA treatment for 8 h plus CHX pretreatment. Error bars represent SD (*n* = 5–8). Significant differences are denoted by letters (Duncan multiple-comparisons test; values within the column followed by the same letter show no significant difference (*p* > 0.05), whereas those with different letter indicate significant difference (*p* < 0.05)).

**Figure 6:**
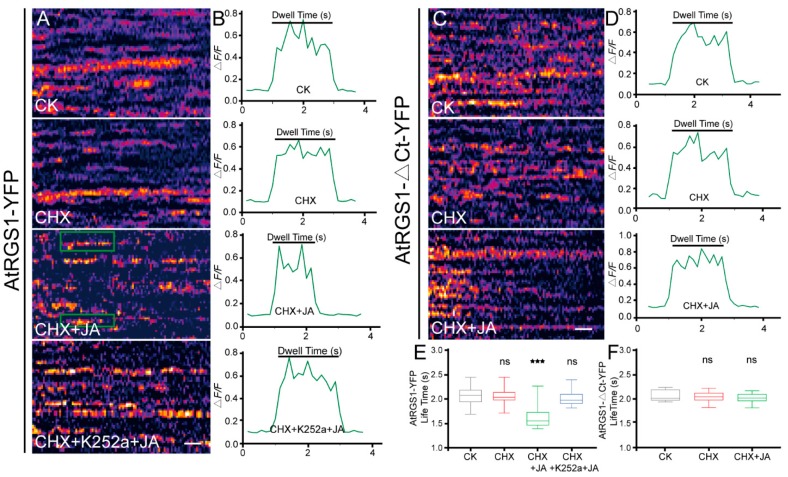
JA treatment affects the dwell time of AtRGS1. (**A**) and (**C**), Representative kymograph showing individual AtRGS1-YFP (**A**) and AtRGS1-ΔCt-YFP (**C**) dwell times without treatment (CK), with CHX treatment, with MeJA treatment for 8 h plus CHX pretreatment, and with MeJA treatment for 8 h plus CHX and K252a pretreatment. Bar = 0.5 s. (**B**) and (**D**), Representative traces of normalized fluorescence of AtRGS1-YFP (**B**) and AtRGS1-ΔCt-YFP (**D**) under different conditions as (**A**) and (**C**) by MATLAB analysis. (**E**,**F**), The average dwell time of AtRGS1-YFP (**E**) and AtRGS1-ΔCt-YFP (**F**) under the different treatment as (**A**) and (**C**). Error bars represent SD (*n* = 10). Significant differences are denoted by asterisks (*** *p* < 0.001, ns, no significant difference, Student’s *t* test).

To determine whether the phosphorylation state of AtRGS1 is required for its endocytosis upon JA treatment, we employed the serine/threonine protein kinases inhibitor, K252a. Similar to our previous observation, AtRGS1-YFP displayed an obvious intracellular accumulation in response to MeJA and CHX co-treatment (Figure 5A). In contrast, a few intracellular puncta were observed in AtRGS1-YFP seedlings after incubation of MeJA with pretreatment of CHX plus K252a (Figure 5A). Statistical analysis of the internalized AtRGS1-YFP fluorescence percentage also showed almost 50% inhibition of AtRGS1-YFP by co-treatment with MeJA, CHX, and K252a compared to that without K252a addition (Figure 5B). We next tested the effect of K252a on the dwell time of AtRGS1 in response to JA treatment by kymograph analysis. With reduced endocytosis, K252a application led to a significantly longer PM lifetime for AtRGS1-YFP upon JA treatment than that without the specific inhibitor (*t* = 1.93 s vs. *t* = 1.56 s) (Figure 6A,B,E). Thus, phosphorylation is an essential step in JA-induced AtRGS1 endocytosis.

To investigate the role of the C-terminal domain of the AtRGS1 protein in its endocytosis upon JA stimulation, we used a C-terminal truncation mutant AtRGS1-ΔCt-YFP assay (Figure S1). As shown in Figure 5C, MeJA did not induce significant internalization of AtRGS1-YFP when the C-terminal domain was truncated, whereas the full-length AtRGS1 induced an increase in the number of fluorescent spots in the cytoplasm at the same time. Quantification assays confirmed that JA treatment induced a two-fold increment in AtRGS1 internalization but not in that of AtRGS1-ΔCt-YFP (Figure 5D). We next examined the effect of C-terminal truncation on its surface lifetime exposed to JA treatment. In contrast to the relative low stability of AtRGS1-YFP, the life time of AtRGS1-ΔCt-YFP was much longer (*t* = 1.56 s vs. *t* = 2.07 s) (Figure 6C,D,F). These results indicate that the C-terminal domain on AtRGS1 is necessary for JA-induced internalization.

Based on these data, we provide compelling evidence to support that both phosphorylation and C-terminal domain are prerequisites for JA-induced endocytosis of AtRGS1. AtRGS1 has a C terminus highly enriched in serine residues that are analogous to phosphorylated sequence of GPCRs [6], some of which are phosphorylated upon activation by d-glucose and flg22 for internalization [15,16]. These findings prompt us to propose that JA mediates endocytosis of AtRGS1 via the phosphorylation of its C-terminal region. However, identification of the candidate kinase and phosphorylated sites is warranted.

### 2.4. JA Treatment Affects PM Dynamics of AtRGS1 and AtRGS1-ΔCt

PM proteins are not static; instead, they exhibit dynamic behaviors. Except for subcellular trafficking, changes in the diffusion dynamics within the PM also occur when cells sense an extracellular signal. Single-particle tracking (SPT) techniques could trace the movements of single protein particles from sequential images by VA-TIRFM. Thus, various diffusion properties of PM proteins in plant cells, such as velocity, trajectory, diffusion coefficient, and mean square displacement, have been quantified by SPT analysis with a high spatiotemporal resolution [21]. Herein, using VA-TIRFM and SPT analysis, we quantitated the diffusion coefficient and motion range of AtRGS1-YFP and AtRGS1-ΔCt-YFP, which reflected their lateral mobility within the PM.

**Figure 7:**
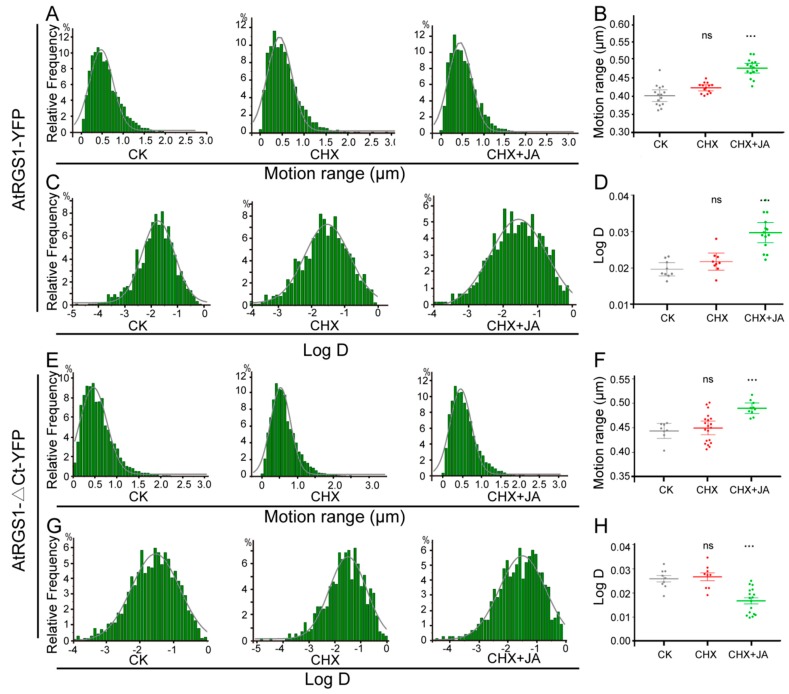
JA treatment affects PM dynamics of AtRGS1 and AtRGS1-ΔCt. (**A**,**E**), Distribution of the motion range of AtRGS1-YFP (**A**, *n*= 12589) and AtRGS1-ΔCt-YFP (**E**, *n*= 12776) without treatment (CK), with CHX treatment, and with MeJA treatment for 8 h plus CHX pretreatment. (**B**,**F**), The average frequency of the AtRGS1-YFP and AtRGS1-ΔCt-YFP motion range under different conditions as (**A**,**E**). Error bars represent SD (*n* = 8–20). Significant differences are denoted by asterisks (*** *p* < 0.001, ns, no significant difference, Student’s *t* test). (**C**,**G**): Distribution of the diffusion coefficients of AtRGS1-YFP (**C**) and AtRGS1-ΔCt-YFP (**G**) without treatment (CK), with CHX treatment, and with MeJA treatment for 8 h plus CHX pretreatment. (**D**,**H**): The average frequency of AtRGS1-YFP and AtRGS1-ΔCt-YFP diffusion coefficients under different conditions as (**C**,**G**). Error bars represent SD (*n* = 8–20). Significant differences are denoted by asterisks (*** *p* < 0.001, ns, no significant difference, Student’s *t* test).

As shown in Figure 7A,C, the motion ranges and diffusion coefficients of AtRGS1-YFP always yielded a single population with different treatments. No distinct differences were found between seedlings without treatment (CK) and with CHX treatment only, whereas the dynamic behaviors of AtRGS1-YFP particles triggered by MeJA significantly changed (Figure 7B,D). For motion range, we found an overall increase after JA treatment, with Ĝ from 0.40 μm (CK) to 0.49 μm (Figure 7B). Moreover, the diffusion coefficient of AtRGS1-YFP upon JA stimulation was markedly higher than that under the non-treated condition (CK) (Ĝ = 2.97 ± 0.18 × 10^–3^ μm^2^/s vs. 1.97 ± 0.08 × 10^–3^ μm^2^/s) (Figure 7D). Taken together, AtRGS1 proteins exhibit comparable trends to increased lateral mobility within the PM in response to JA treatment.

Parallel experiments were also performed on AtRGS1-ΔCt-YFP. Consistent with AtRGS1-YFP, the motion ranges and diffusion coefficients of AtRGS1-ΔCt-YFP were also characterized by a single population (Figure 7E,G). Seedlings without treatment (CK) and treated with CHX only showed similar dynamic features (Figure 7F,H). Compared to the non-treated condition (CK), there was a distinct trend towards an increased motion range (Ĝ = 0.44 μm vs. Ĝ = 0.49 μm) and decreased diffusion coefficient (Ĝ =2.59 ± 0.08 × 10^–3^ μm^2^/s vs. 1.68 ± 0.17 × 10^–3^ μm^2^/s) (Figure 7F,H). These observations suggest that JA treatment leads to a longer motion trajectory and lower diffusion coefficient of AtRGS1-ΔCt.

Collectively, full-length AtRGS1 and C-terminus-truncated AtRGS1 proteins had similar variations in motion range upon JA treatment and showed the opposite behavior for diffusion coefficients. Dynamic analysis of these molecules as individual entities will shed light on the underlying mechanisms of G protein signaling.

## 3. Materials and Methods

### 3.1. Plant Materials and Growth Conditions

*Arabidopsis thaliana* ecotype Columbia-0 (Col-0) was used as a wild-type control. The *gpa1-4*, *agb1-2*, and *gpa1-4/agb1-2* mutants [15], and the 35S::AtRGS1-YFP [15] and 35S::AtRGS1-ΔCt-YFP [16] transgenic lines were kindly provided by Dr. Alan M. Jones, University of North Carolina at Chapel Hill (Chapel Hill, NC, USA). Seeds were surface-sterilized with ethanol and H_2_O_2_ mixture (70% ethanol:30% H_2_O_2_ = 4:1), and then stratified at 4 °C for 3 days in the dark. For root length measurements, anthocyanin measurement, and real-time PCR analysis, seeds were plated and grown on ½ MS medium supplemented with 1% sucrose under a 16-h light (23–25 °C)/8-h dark (17–20 °C) photoperiod. For confocal and VA-TIRFM analyses, seeds were sown on liquid ½ MS medium without sucrose, followed by 2 h light, and then grown in the dark for 3 days followed by drug treatment and imaging.

### 3.2. Anthocyanin Measurements

The anthocyanin quantification of 11-day-old *Arabidopsis* seedlings grown on MS medium with 0 or 25 mM MeJA was performed as previously described [22]. Anthocyanin content was reported as (A535–A650)/g fresh weight. For each treatment, the experiment was repeated thrice.

### 3.3. Root Length Measurement

The primary root length of 9-day-old *Arabidopsis* seedlings grown on MS medium with 0, 10, and 25 mM MeJA was measured by ImageJ software (Image high-energy version 1.4.3.67, National Institutes of Mental Health, Bethesda, MD, USA). For each treatment, the experiment was repeated thrice.

### 3.4. Quantitative Real-Time PCR Analysis

Three-week-old plants grown on MS medium were drenched in solution containing 100 mM MeJA or water for 8 h in the daytime and then harvested. Total RNA extraction and cDNA synthesis were conducted with an RNA prep Pure Plant Kit (Tiangen Biotech Co., Ltd., Beijing, China) and PrimeScript 1st Strand cDNA Synthesis Kit (Takara Bio Co., Ltd., Shiga, Japan), respectively. Quantitative real-time PCR analysis was performed with the Bio-Rad CFX Connect real-time PCR system (Bio-Rad, Hercules, CA, USA) by SYBR Premix Ex Taq II (Tli RNase H Plus) (Takara Bio Co., Ltd., Shiga, Japan). *ACTIN1* served as the internal control. Gene-specific primers are listed in Table S1. For each treatment, the experiment was repeated thrice.

### 3.5. Transient Infiltration of N. benthamiana Leaves

The AtRGS1-GFP and AtGPA1-mCherry plant expression constructs were developed by inserting the AtRGS1 and AtGPA1 coding sequence without a stop codon to pCAMBIA2300-35S-GFP and pCAMBIA1300-35S-mCherry vectors, respectively. The primers are shown in Table S1. Leaves of seven-week-old *N. benthamiana* were co-infiltrated with equal volumes of different combinations of *Agrobacterium* strains (AtRGS1-GFP with AtGPA1-mCherry). Plants were maintained at 22 °C for two days in the dark before imaging.

### 3.6. Drug Treatment

Dark-grown 3-day-old seedlings were incubated in deionized water containing the indicated compounds for the following times and concentrations: 4–10 h for 50 μM CHX; 2 h pretreatment with 50 μM CHX and then 2–8 h for 100 μM MeJA plus CHX; and 2 h pretreatment with 50 μM CHX, 30 min pretreatment with 10 μM K252a, and then 8 h for 100 μM MeJA plus CHX and K252a.

### 3.7. Confocal Microscopy

A Zeiss LSM710 confocal laser scanning microscope with a C-Apochromat 40/1.2 water immersion objective was used to image the vertical optical sections (Z stacks) of hypocotyl epidermal cells located 2 to 4 mm below the cotyledons. The YFP fluorescence signals were excited by a 514 nm laser line and were collected at 526 to 569 nm by the photomultiplier detector. A series of Z-section images were acquired from the top layer of cells with 0.5-μm steps. Images at 2 to 3 μm below the apical plasma membrane were used to quantify the internalization of AtRGS1-YFP or AtRGS1-ΔCt-YFP by the software ImageJ (Image high-energy version 1.4.3.67, National Institutes of Mental Health, Bethesda, MD, USA). For internalization analysis, the ratio between the fluorescence signal intensity in the cytoplasm and the whole cell represented the fraction of internalized AtRGS1-YFP or AtRGS1-ΔCt-YFP. For each treatment, the experiment was repeated thrice.

### 3.8. VA-TIRFM and Fluorescence Image Analysis

The epidermal cells of hypocotyl were imaged on an Olympus microscope using a 100× 1.45 NA oil immersion objective. AtRGS1-YFP or AtRGS1-ΔCt-YFP proteins were excited with a 488 nm laser line from a diode laser (Chang-chun New Industries Optoelectronics Technology), and the emission fluorescence was collected with an EM-CCD camera (ANDOR iXon DV8897D-CS0-VP, Andor Technology, Belfast, UK) using bandwidth filters ranging from 505 to 540 nm. Images were acquired with 150 ms exposure times and a time-lapse series of single particles of AtRGS1-YFP was taken with up to 100 images per sequence. The stand-alone MATLAB (R2014) Graphical User Interface program was used for SPT analysis according to the method described by Jaqaman [23]. Kymograph and lifetime analyses were performed as previously described [24]. After tracking the trajectories of individual particles, the mean square displacement (MSD) was computed [25]. The diffusion coefficient of a specific particle was calculated by linear fit to MSD versus time (MSD-t) plots [25]. The motion range was determined as the largest displacement during the lifetime [25]. The distribution of the diffusion coefficients or motion range was plotted in a histogram [25]. The data were fitted by a Gaussian function, and the position of the peak (denoted as Ĝ) was considered as characteristic values for further analysis [25]. For each treatment, the experiment was repeated twice.

### 3.9. FLIM-FRET Measurement

FLIM was performed on an inverted Olympus FV1200 microscope equipped with a PicoQuant picoHarp300 controller (PicoQuant, Berlin, Germany). Excitation at 488 nm was conducted with a picosecond pulsed diode laser, and the emitted light was filtered with a 505/540 nm bandpass filter and detected with an MPD SPAD detector. Single-pixel fluorescence lifetimes were averaged across a representative region of interest (ROI). The resulting images showed the corresponding fluorescence lifetime, τ, for each pixel in a rainbow color code from navy (τ = 2.1 ns) to red (τ = 2.7 ns). FRET efficiency (%) was calculated as described previously [26]. For each treatment, the experiment was repeated twice.

### 3.10. Western Blot Analysis

Total proteins were extracted from the transgenic seedlings expressing AtRGS1-YFP and AtRGS1-ΔCt-YFP. The amount of AtRGS1 and AtRGS1-ΔCt proteins was determined using a specific anti-GFP antibody (Sigma, St.Louis, MI, USA).

### 3.11. Data Analysis

Significant differences are denoted by letters (*p* < 0.05; Duncan multiple-comparison test) and by asterisks (* *p* < 0.05, *** *p* < 0.001, ns, not significant; Student’s *t* test). The standard deviation (SD) was used to calculate the error bars.

### 3.12. Accession Numbers

This data used in this study are available from the *Arabidopsis* Information Resource (TAIR) database under accession numbers AT3G26090.1 (AtRGS1), AT2G26300 (AtGPA1), AT4G03415 (AGB1), AT5G24780 (VSP1), and AT3G45140 (LOX2).

## 4. Conclusions

In conclusion, these results demonstrate that the G protein-mediated pathway is involved in JA signaling, which possibly requires G protein activation via phosphorylation and C-terminus-dependent AtRGS1 endocytosis indirectly by JA.

## Supplementary Materials

Supplementary materials can be found at https://www.mdpi.com/1422-0067/20/15/3779/s1. Figure S1: Western blot analysis of the AtRGS1-YFP and AtRGS1-ΔCt-YFP; Figure S2: Co-transient expression of AtRGS1-GFP and AtGPA1-mCherry in *N. benthamiana* leaves; Table S1: Primers used in this study.
