# Supplementary figures and images for: JA-Induced Endocytosis of AtRGS1 Is Involved in G-Protein Mediated JA Responses
Source: Int J Mol Sci. 2019 Aug 2;20(15):3779. doi: 10.3390/ijms20153779 (PMC6695760; doi:10.3390/ijms20153779)

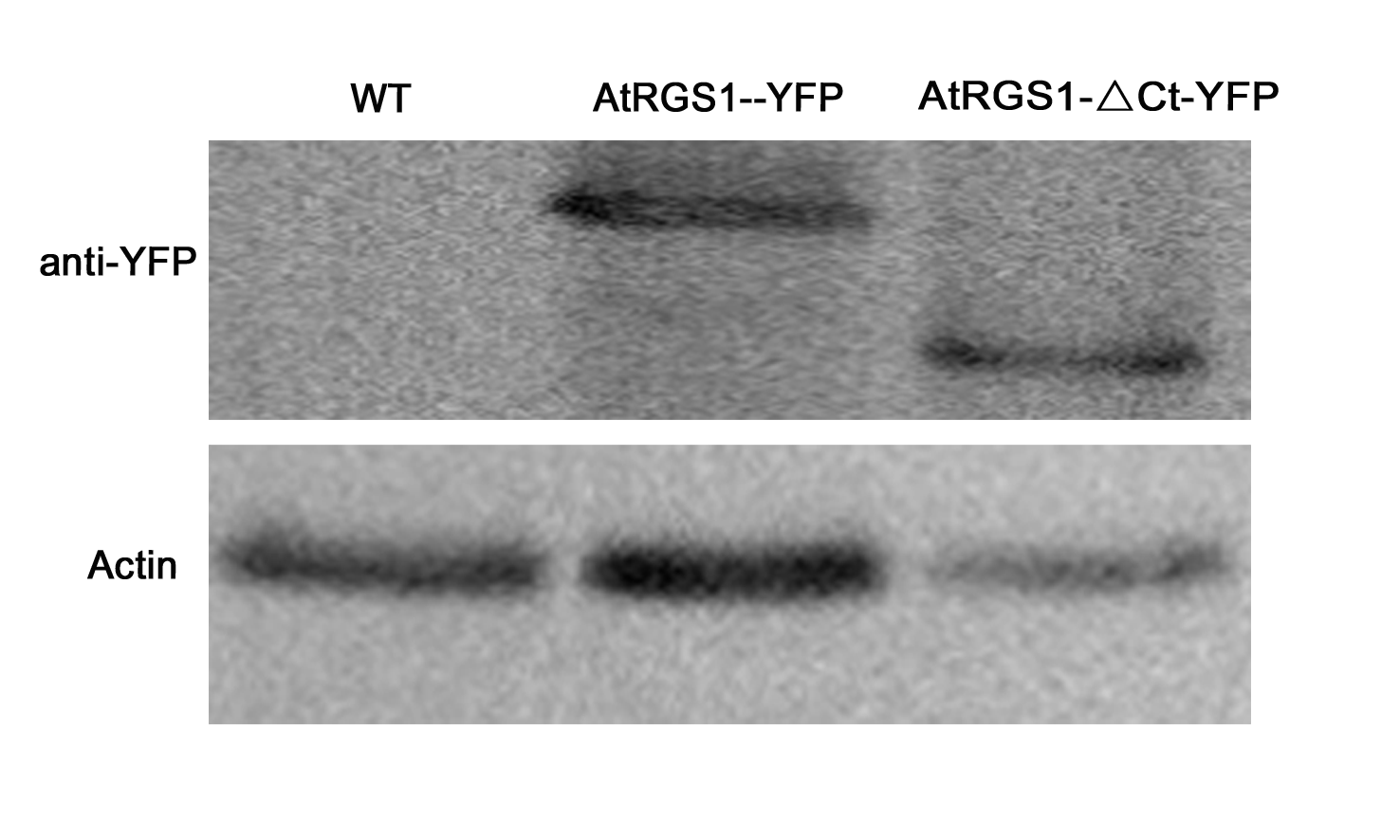

Supplement: Supplementary file 1 [file ijms-20-03779-s001.zip › Figure S1.tif]

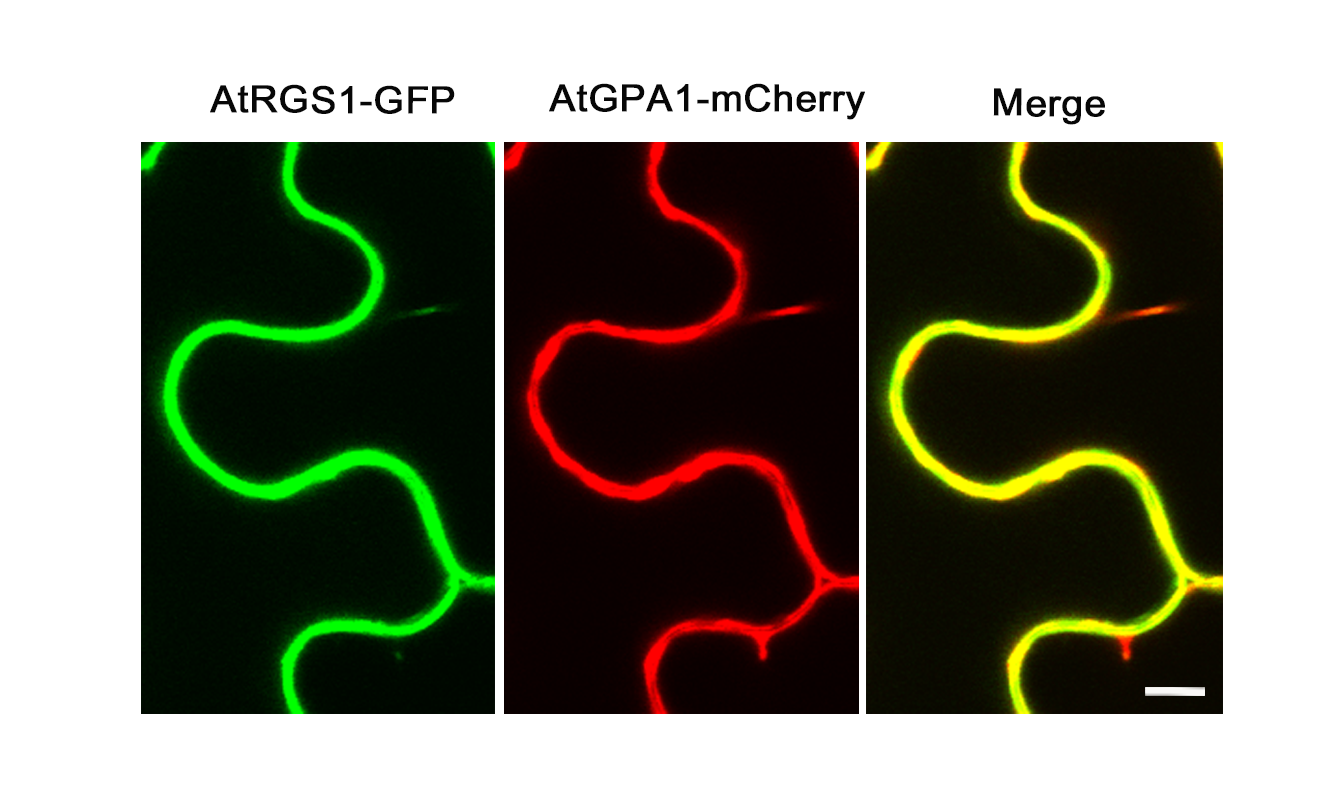

Supplement: Supplementary file 1 [file ijms-20-03779-s001.zip › Figure S2.tif]

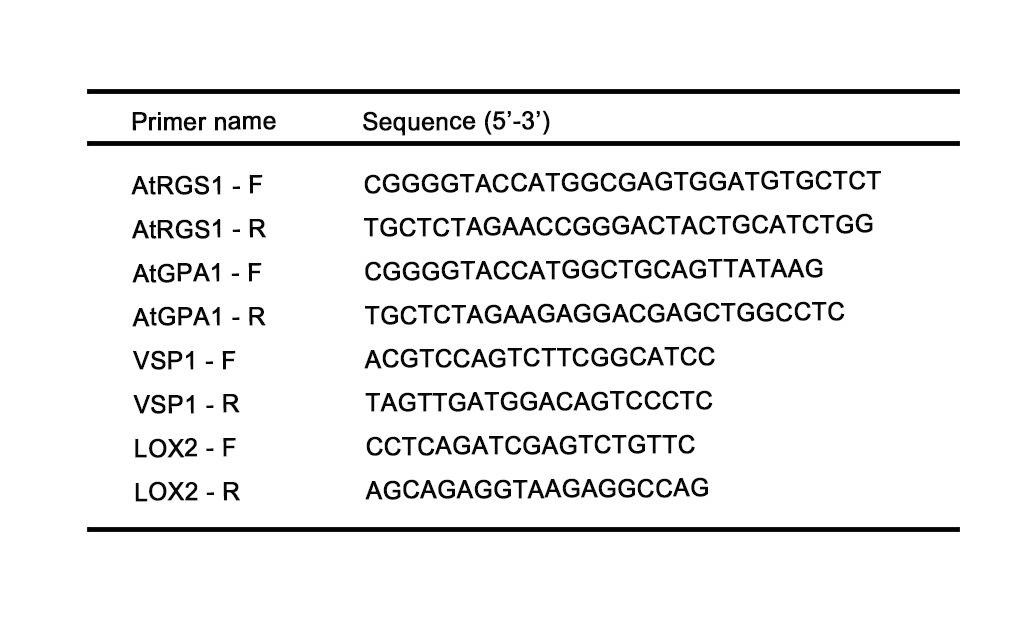

Supplement: Supplementary file 1 [file ijms-20-03779-s001.zip › Table S1.tif]
